# Development and validation of a classification and scoring system for the diagnosis of oral squamous cell carcinomas through confocal laser endomicroscopy

**DOI:** 10.1186/s12967-016-0919-4

**Published:** 2016-06-03

**Authors:** Nicolai Oetter, Christian Knipfer, Maximilian Rohde, Cornelius von Wilmowsky, Andreas Maier, Kathrin Brunner, Werner Adler, Friedrich-Wilhelm Neukam, Helmut Neumann, Florian Stelzle

**Affiliations:** Department of Oral and Maxillofacial Surgery, Friedrich-Alexander University Erlangen-Nürnberg (FAU), University Hospital Erlangen, Glückstraße 11, 91054 Erlangen, Germany; Department of Computer Science 5, Friedrich-Alexander University Erlangen-Nürnberg (FAU), Martensstraße 3, 91058 Erlangen, Germany; Institute of Pathology, Friedrich-Alexander University Erlangen-Nürnberg (FAU), Krankenhausstr. 8-10, 91054 Erlangen, Germany; Department of Medical Information Technology, Biometry and Epidemiology, Friedrich-Alexander University Erlangen-Nürnberg (FAU), Waldstraße 6, 91054 Erlangen, Germany; Department of Medicine I, Friedrich-Alexander University Erlangen-Nürnberg (FAU), University Hospital Erlangen, Ulmenweg 18, 91054 Erlangen, Germany; SAOT-Erlangen Graduate School in Advanced Optical Technologies, Friedrich-Alexander University Erlangen-Nürnberg (FAU), Paul Gordan Strasse 6, 91052 Erlangen, Germany

**Keywords:** Confocal laser endomicroscopy, CLE, Oral squamous cell carcinoma, OSCC, Oral cancer, DOC-score, Real-time histology, Optical biopsy

## Abstract

**Background:**

Confocal laser endomicroscopy (CLE) is an optical biopsy method allowing in vivo microscopic imaging at 1000-fold magnification. It was the aim to evaluate CLE in the human oral cavity for the differentiation of physiological/carcinomatous mucosa and to establish and validate, for the first time, a scoring system to facilitate CLE assessment.

**Methods:**

The study consisted of 4 phases: (1) CLE-imaging (in vivo) was performed after the intravenous injection of fluorescein in patients with histologically confirmed carcinomatous oral mucosa; (2) CLE-experts (n = 3) verified the applicability of CLE in the oral cavity for the differentiation between physiological and cancerous tissue compared to the gold standard of histopathological assessment; (3) based on specific patterns of tissue changes, CLE-experts (n = 3) developed a classification and scoring system (DOC-Score) to simplify the diagnosis of oral squamous cell carcinomas; (4) validation of the newly developed DOC-Score by non-CLE-experts (n = 3); final statistical evaluation of their classification performance (comparison to the results of CLE-experts and the histopathological analyses).

**Results:**

Experts acquired and edited 45 sequences (260 s) of physiological and 50 sequences (518 s) of carcinomatous mucosa (total: 95 sequences/778 s). All sequences were evaluated independently by experts and non-experts (based on the newly proposed classification system). Sensitivity (0.953) and specificity (0.889) of the diagnoses by experts as well as sensitivity (0.973) and specificity (0.881) of the non-expert ratings correlated well with the results of the present gold standard of tissue histopathology. Experts had a positive predictive value (PPV) of 0.905 and a negative predictive value (NPV) of 0.945. Non-experts reached a PPV of 0.901 and a NPV of 0.967 with the help of the DOC-Score. Inter-rater reliability (Fleiss` kappa) was 0.73 for experts and 0.814 for non-experts. The intra-rater reliability (Cronbach’s alpha) of the experts was 0.989 and 0.884 for non-experts.

**Conclusions:**

CLE is a suitable and valid method for experts to diagnose oral cancer. Using the DOC-Score system, an accurate chair-side diagnosis of oral cancer is feasible with comparable results to the gold standard of histopathology—even in daily clinical practice for non-experienced raters.

## Background

According to the GLOBOCAN series for the *International Agency for Research on Cancer*, there is an incidence of 14.1 million new cases of cancer worldwide per year. This number is expected to rise substantially over the next years [1]. Ten percent of all cancer cases are neoplasms of the head and neck area, of which more than 90 % are classified as squamous cell carcinomas (HNSCCs) [2, 3]. Main risk factors for Squamous Cell Carcinomas (SCC) of the oral cavity are the consumption of tobacco or alcohol [4]. In recent times, HNSCCs with a Human Papilloma virus infection, especially HPV 16, have been increasing [5] and represent about 20–25 % of all HNSCC cases [6]. Consequently, not only high-risk groups associated with alcohol and tobacco use but also a growing group of younger patients with HPV-infections are affected by cancer in the oral cavity.

Even though the therapy of HNSCCs has improved substantially—regarding the advancement in surgical methods and the development of specific radio and chemotherapy regimens over the last decades—one of the most crucial factors in the successful treatment of oral cancer still remains the early and accurate diagnosis [7–9].

The present gold standard for the diagnosis of oral malignant neoplasms starts with a visual gross identification of SCCs and its precursor lesions, followed by an invasive biopsy and a histopathological assessment [8]. The initial visual examination of the oral cavity is regarded as highly subjective, depends on the examiner’s clinical experience and requires specific training [5, 10]. The subsequent biopsy and histopathological tissue analysis are time and resource consuming and can also have individual variances in diagnosis [11, 12].

Optical imaging methods have the potential to fill the gap in daily clinical practice between the subjective visual examination and the invasive biopsy followed by a histopathological assessment. Confocal laser endomicroscopy (CLE) was recently introduced as a novel technique allowing in vivo microscopic evaluation of the tissue during the ongoing examination in real time by providing an optical biopsy [3, 13]. This biomedical method could enable an in vivo diagnosis and can be reproduced almost indefinitely without iatrogenic damage to the patient and the need for additional human resources.

Recently, CLE has moved from a research tool to a valuable diagnostic technique in medical disciplines like gastroenterology [14–18]. Also preliminary results for a CLE diagnosis of oral cancer are promising [3, 13, 19–26]. Transferring first experimental results of CLE in the human oral cavity into a valid and evidence based clinical setting is an essential step for this imaging tool on its way from bench to bedside [27].

The aim of our study was to depict the applicability of CLE examinations for diagnosing oral squamous cell carcinomas (OSCCs) in comparison to the gold standard of histopathological examination. Furthermore, we developed and evaluated, for the first time in this specialty and localization, an easy-to-use and straightforward scoring system for the identification of OSCCs on the basis of morphological and architectural criteria in order to enable a valid and reproducible CLE assessment even for non-experts in their daily clinical practice.

## Methods

### Study design and setting

An experimental clinical study was carried out to evaluate the clinical benefit of CLE assessment and furthermore to develop and validate a classification and scoring system for the diagnosis of squamous cell carcinoma of the oral cavity by using CLE with intravenous (IV) fluorescent dye. Prior to examinations, Institutional Review Board (IRB) approval was obtained (ethics committee of the University of Erlangen-Nürnberg; reference number: 243_12 B).

The study consisted of four phases: Phase 1, CLE Imaging. Phase 2, validation of the classification performance by experts (n = 3) in differentiating between physiological and cancerous tissue with the help of CLE and comparison to histopathological analyses. Phase 3, analysis of collected visual material and development of a classification and scoring system by CLE-experienced specialists. Phase 4, validation of the scoring system by non-experts in the field of CLE (n = 3) through assessment of the reliability in predicting the histopathological classification. Comparison of these results with the outcomes by experts and the gold standard of histopathology. Evaluation of the inter- and intra-rater reliability.

### Patients

Our study is a survey of all inpatients of the Oral and Maxillofacial Surgery Department of the Erlangen University Hospital with histopathologically confirmed oral squamous cell carcinoma from 5/2013 to 7/2015. Exclusion criteria were defined as follows: age <18 years; current pregnancy/lactation period; allergies to fluorescein; renal insufficiency at any stage; beta-blocker medication. All lesions were histologically verified by the gold standard of biopsy and histopathological assessment by a pathologist specialized in cancer of the head and neck region, not involved in the CLE imaging.

### Examiners

Two groups of independent examiners participated in the rating of CLE video sequences. The first group consisted of three medical experts with prior CLE experience. The second group (dentistry fellows; n = 3) served as non-experts with no prior experience in CLE imaging.

### CLE—experimental setup and dye used

Video sequences were recorded using a stand-alone probe-based CLE system (pCLE: ColoFlex UHD Probe, Cellvizio, Mauna Kea Technologies, Paris, France). This system offers a video frame rate of 12 frames/s, an image size of 576 × 578 pixels and a maximum penetration depth of 65 µm. The field of view has a diameter of 240 µm. Since normal tissue background fluorescence is not able to generate a sufficient signal-to-noise ratio [28], intravenous fluorescent dye was applied (fluorescein Alcon^®^ 10 %). An initial dose of 3 mL prior to the examination with a maximum dose of 7.5 mL of fluorescein (IV) during the measurement procedure was defined according to experiences of our workgroup in the gastrointestinal tract.

### Procedure

#### Phase 1: CLE imaging

All patients were informed about potential risks and signed an informed consent form prior to the study according to the IRB approval of the ethics committee of Friedrich-Alexander-University of Erlangen-Nürnberg (FAU, Approval Number 243_12 B). Examinations were performed on conscious patients and were totally integrated into the clinical procedure. To enhance image quality, the mouth was rinsed with acetylcysteine solution (ACC 100 mg Hexal, effervescent tablets) by the patients prior to confocal imaging. After the fluorescent agent was injected, the probe was gently applied on the surface mucosa. In total, each patient had four sites imaged and every location was recorded as individual video sequences. Three reference points were determined for CLE imaging of physiological tissue: lower inner labium, upper alveolar ridge and palatal region. Last measured point was the lesion itself (OSCC). CLE imaging of the cancerous tissue was conducted in a standardized manner by focusing on the transition between physiological oral mucosa and the lesion with a subsequent adjustment on the center of the carcinoma.

#### Phase 2: validation of the classification performance in differentiating between physiological and cancerous tissue by CLE examinations

The CLE library was presented to experts (n = 3) independently and blinded in a random manner. They used their own expertise in CLE imaging to classify each sequence as “physiological mucosa” or “oral squamous cell carcinoma”. The final ratings were statistically compared to the present gold standard for the diagnosis of oral cancer (histopathological examination). One of these three examiners evaluated all sequences for a second time 4 weeks later in order to calculate the intra-rater reliability.

#### Phase 3: development of a classification and scoring system

CLE experts (n = 3) examined the footage in order to identify potential classification parameters for SCCs of the oral cavity. They were encouraged to include parameters that are simple, effective and easily applicable in daily clinical practice. The relationship between each defined parameter and the histological findings was modeled and afterwards these criteria were ranked according to their importance in identifying OSCCs. A scoring system was established on the basis of these findings in multiple discussion rounds (*DOC*-*Score*; Table 1).

**Table 1:** DOC-Score (*d*iagnosing *o*ral cancer with the help of *C*LE)

Evaluation criterion	Manifestation	Score
1. Tissue architecture		
a) Homogeneity	Completely organized	0
	Organized + unorganized regions	1
	Completely unorganized	2
b) Intercellular gaps	Regular	0
	Mutated	1
	No longer/non-existent	2
2. Cell morphology	Consistent/regular	0
	Inconsistent/dysplastic (different sizes/shapes/grey levels)	1
	Completely irregular Dark, small cells Blurry, cloudy image Black spot (cell cluster)	2
3. Fluorescence leakage	Regular	0
	Amplified (bright background)	1
4. Vessels	Regular	0
	Irregular Shape (micro vessels, coiled, elongated etc.) Caliber (increased) Quantity (cumulated)	1
*x* ≥ *5 points* = *squamous cell carcinoma (SCC)*		Max. total: 8 points

CLE classification and scoring system of oral squamous cell carcinomas

#### Phase 4: validation of the scoring system by assessing the reliability of predicting the histological classification

**Fig. 1:**
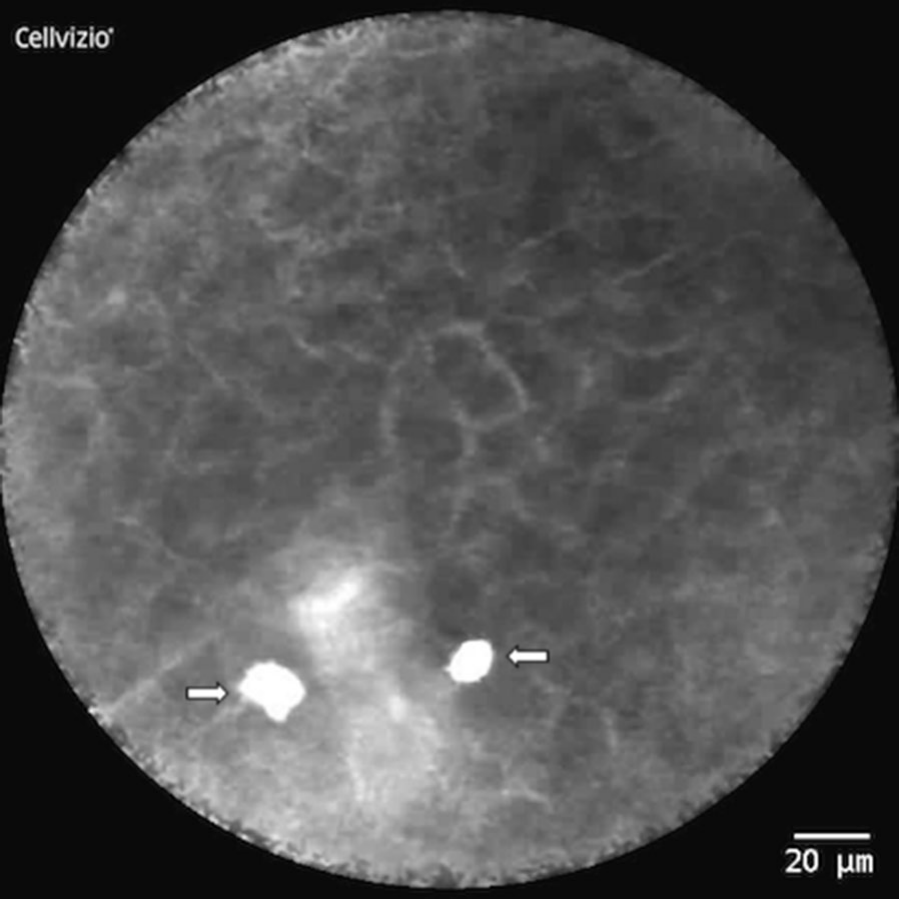
CLE-image of physiological tissue (type of epithelium: attached gingiva; location: alveolar process); homogeneous visual appearance through completely organized tissue architecture (0p.) and slim, accurate intercellular gaps (0p.); consistent cell morphology in shape and color (0p.); no amplified fluorescein leakage (0p.); regular vessels (see *arrows*) (0p.)

**Fig. 2:**
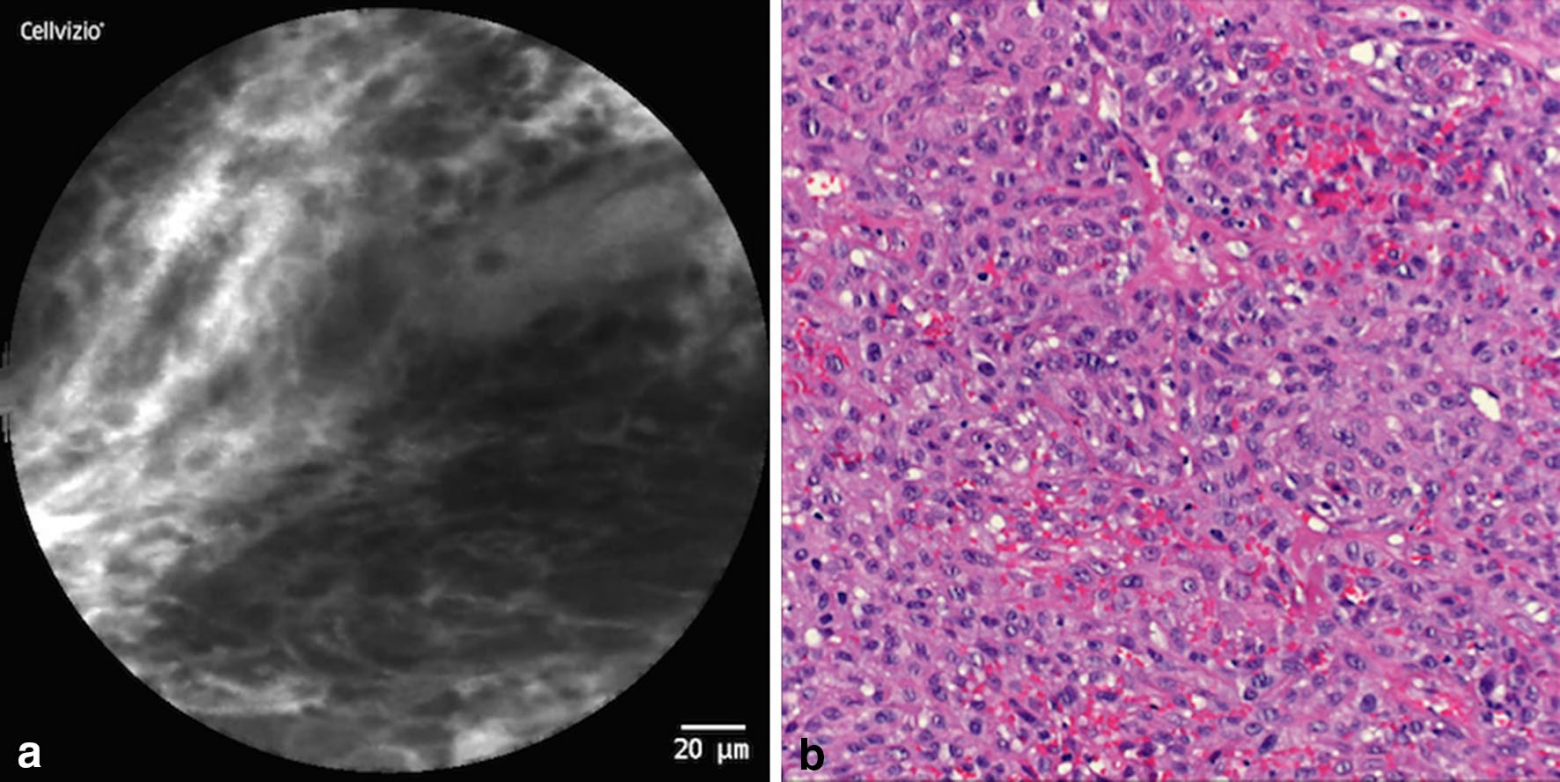
OSCC (location: alveolar process); **a** CLE-Image: Unorganized tissue architecture (2p.); intercellular gaps not definable in some spots (2p.); irregular cells (shape, color and size) and cell cluster (=*black spots*; 2p.); slightly intensified fluorescein leakage (1p.); vessels not assessable (0p.); **b** Micrograph (HE staining) of the corresponding specimen

**Fig. 3:**
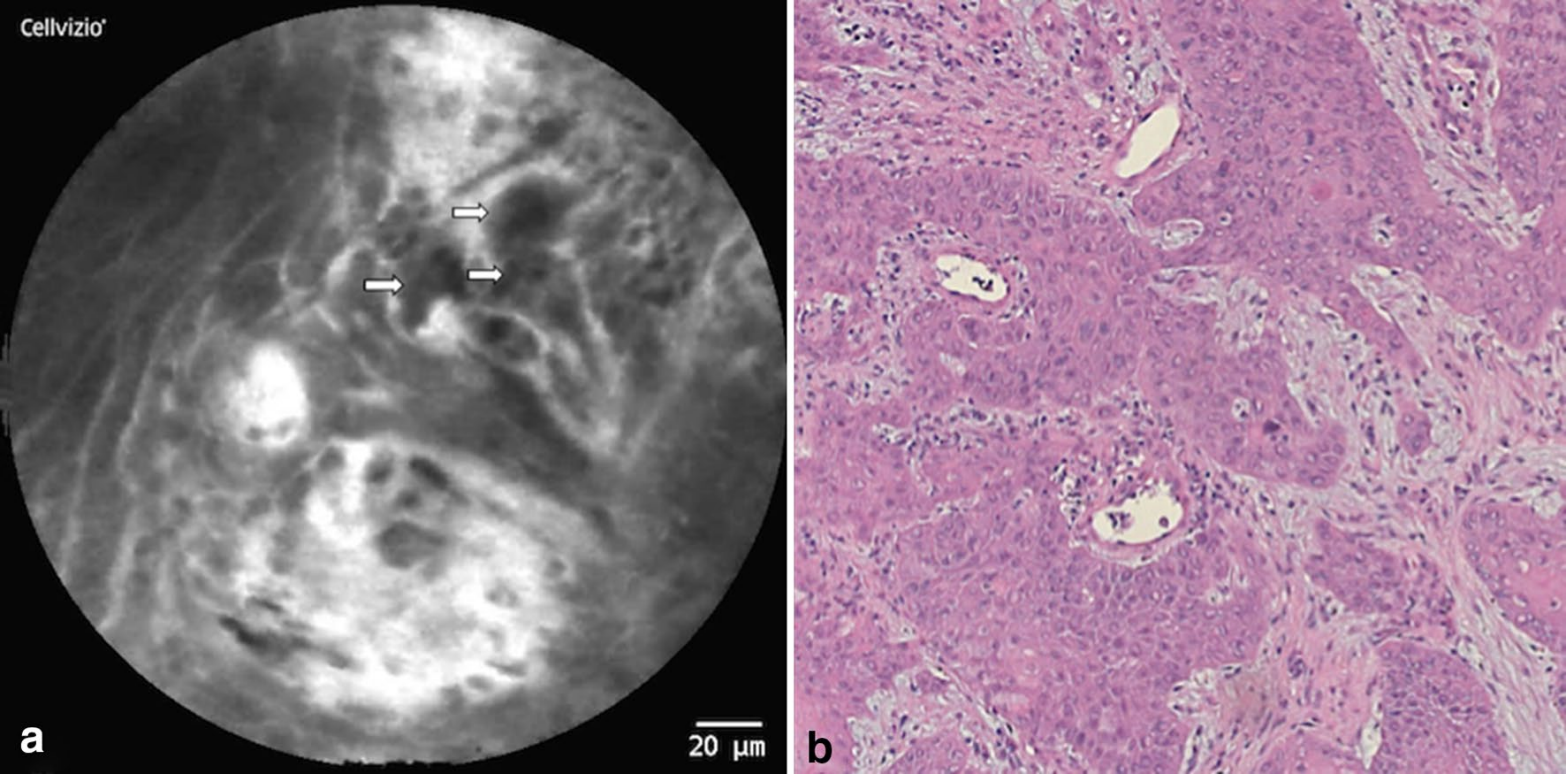
OSCC (location: anterior floor of the mouth); **a** CLE-Image: Unorganized tissue architecture (2p.); non-existent intercellular gaps (2p.); irregular cells like cell clusters (=*black spots*; see *arrows*) (2p.); amplified fluorescein leakage (1p.); no visible vessel changes (0p.); **b** Micrograph (HE staining) of the corresponding specimen

**Fig. 4:**
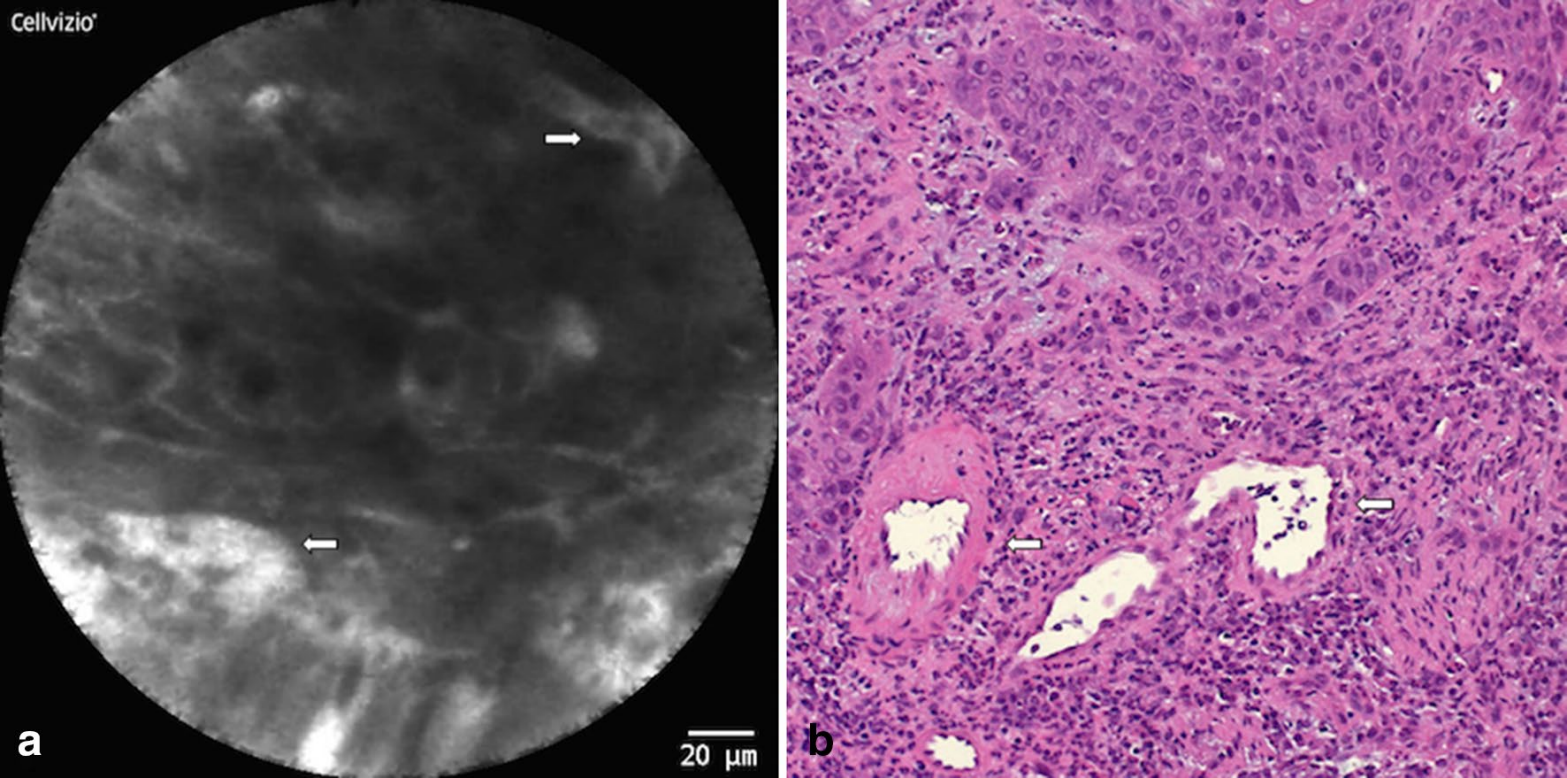
OSCC (location: anterior floor of the mouth): **a** CLE-Image: Completely unorganized tissue architecture (2p.); non-existent intercellular gaps (2p.); blurry, cloudy image because of indistinct cell borders (2p.); no increased fluorescein leakage (0p.); irregular vessels (*coiled*; see *arrows*) (1p.); **b** Micrograph (HE staining) of the corresponding specimen showing similar changes in vessel morphology (see *arrows*), cellular structure and tissue architecture

All video sequences were presented to non-experts (n = 3; dentists) independently and blinded in a random manner. The inexperienced examiners made a classification based on our newly developed scoring sheet (*DOC*-*Score*) after being introduced to the field of CLE imaging by a unit of instruction slides. This unit presented our criteria list and explained each scoring parameter by using typical sample images of physiological tissue (Fig. 1) as well as OSCC images (Figs. 2, 3, 4) and the scoring regimen. Also potential interfering factors like artifacts or mucus at the tip of the probe were discussed in the slides. Each criterion of the questionnaire was individually based on the presence or absence of specific parameters. The sum of all single values was used to decide on a diagnosis of “physiological tissue” or “OSCC”.

The ratings of non-experts were statistically compared with the results of the examinations by experts and with the histopathological assessment. One of these three non-experts assessed all sequences once again 4 weeks later in order to calculate the intra-rater reliability, as well (similar to phase 2). Inter-rater and intra-rater reliability was calculated for each of the two groups (experts and non-experts).

### Statistics

Statistical analyses were conducted with SPSS version 22 (IBM, Armonk, NY, USA). We calculated occurrence frequency, significance (Chi Square) and correlation (Spearman’s rho) with the histopathological result of each component of the evaluation criteria. Sensitivity, specificity and negative and positive predictive values of diagnosing cancer/non-cancer were evaluated with the help of our scoring system (non-experts) or the subjective impression (experts). Inter- and intra-rater reliability were calculated using Fleiss’ kappa and Cronbach’s alpha.

## Results

### Phase 1: CLE imaging

A total of 95 sequences were acquired (11 sequences of the labium, 20 sequences of the alveolar ridge, 14 sequences of the palatal region and 50 sequences of cancerous tissue). The initial CLE library was reduced to representative video sequences (n = 95) with durations between 1 and 42 s (total 778 s) in order to remove artifacts and blind parts (physiological oral mucosa: 45 sequences/in total 260 s/median: 4 s per sequence; squamous cell carcinoma and carcinoma in situ: 50 sequences/in total 518 s/median: 6,5 s per sequence).

### Phase 2: validation of the classification performance in differentiating between physiological and cancerous tissue through CLE examinations

The classification performance of the experts (n = 3) is shown in Table 2 (individual and averaged performance). Sensitivity and specificity in identifying OSCC as well as the positive predictive and negative predictive value for predicting a histopathological OSCC diagnosis were calculated for each expert. The averaged sensitivity of experts in diagnosing OSCCs was 0.953, whereas specificity yielded a value of 0.889. The weighted Fleiss´ kappa coefficient for multi-rater analysis was calculated (Table 3). With a value of 0.73, substantial inter-individual agreement in diagnosing OSCCs through CLE sequences was shown. Additionally one expert subjectively rated the CLE sequences after a delay of 4 weeks. The intra-individual absolute agreement measure (Table 4) was excellent with a Cronbach’s alpha of 0.989 and an intraclass-correlation coefficient of 0.979 (95 % confidence interval between 0.969 and 0.986).

**Table 2:** CLE classification performance of the experts (subjective evaluation)

Expert	Sensitivity %	Specificity %	PPV %	NPV %
1	100 (50/50)	95.6 (43/45)	96.2 (50/52)	100 (43/43)
2	86.0 (43/50)	97.8 (44/45)	97.7 (43/44)	86.3 (44/51)
3	100 (50/50)	73.3 (33/45)	80.6 (50/62)	100 (33/33)
Averaged	95.3 (143/150)	88.9 (120/135)	90.5 (143/158)	94.5 (120/127)

*PPV* positive predictive value, *NPV* negative predictive value

**Table 3:** Inter-rater reliability

Examiner	Fleis’ kappa	Agreement	
		Expected	Observed
Experts (n = 3)	0.730	0.506	0.867
Non-experts (n = 3)	0.814	0.507	0.909

Inter-rater reliability of experts (subjective evaluation) and non-experts (using DOC-score for diagnosis) in CLE assessment

**Table 4:** Intra-rater reliability

Examiner	Reliability; Cronbach’s alpha	Intraclass correlation; (95 % confidence interval)
Expert (n = 1)	0.989	0.979 (0.969–0.986)
Non-expert (n = 1)	0.884	0.792 (0.703–0.856)

Comparison of first and second assessment (video sequences; n = 95) executed by one expert (subjective evaluation) and one non-expert (scoring system) with a delay of 4 weeks

### Phase 3: development of a classification and scoring system

First, experts (n = 3) identified specific parameters that are representational of architectural and morphological tissue changes in CLE imaging. The occurrence frequency of these parameters in physiological as well as in carcinomatous CLE images is shown in Table 5. Subsequently, these items were tested according to their individual classification performance. The significance and correlation coefficients of the bivariate correlation model (score item—histo-pathological gold standard) is also shown in Table 5. Each of the score sheet items showed a significant correlation of p < 0.001 with coefficients ranging from 0.557 (fluorescein leakage) to 0.936 (homogeneity). Chi Square modeling for each item against histopathological diagnosis yielded a highly significant difference between the expected values and the observed data in each case (p < 0.001). According to that, a score sheet was developed considering all important morphometric observations by experts as well as the statistical coefficient values.

**Table 5:** Classification performance of the chosen parameters for the score sheet (by experts)

Parameters (score sheet)	Occurrence frequency, %; anomalies in		Chi square test (n = 95)	Correlations (n = 95)	
	Physiological mucosa (n = 45)	OSCC (n = 50)	P value	P value (2 tailed)	Spearman’s rho
Homogeneity	15.6 (7/45)	100 (50/50)	<0.001	<0.001	0.936
Intercellular gaps	37.8 (17/45)	100 (50/50)	<0.001	<0.001	0.880
Cell morphology	28.9 (13/45)	100 (50/50)	<0.001	<0.001	0.949
Fluorescein leakage	22.2 (10/45)	78 (39/50)	<0.001	<0.001	0.557
Vessel morphology	17.8 (8/45)	92 (46/50)	<0.001	<0.001	0.748

Occurrence frequency of anomalies (mildly or highly dysplastic) in physiological mucosa and OSCCs, Chi square test and Spearman’s correlation

During the evaluation of a CLE sequence, each criterion can be considered and rated separately with a score. Three point values (0 = physiological; 1 = mildly dysplastic; 2 = highly dysplastic) were set for each of the following criteria: homogeneity, intercellular gaps and cell morphology. Fluorescein leakage and vessel morphology scores are represented by two point values (0 = physiological; 1 = dysplastic). The scores are added up to a total sum that has a maximum of eight points. According to the experts’ opinion and their theoretic modeling of the point values for each of the items and the sum score of the sheet, a threshold of five points was identified for the differentiation between physiological and pathological CLE sequences. A point range under 5 indicates the diagnosis of “physiological tissue” (0 to 4 points), whereas a score of 5 points or higher implies the presence of an OSCC (5 to 8 points). The final score sheet is shown in Table 1*(DOC*-*Score)*.

### Phase 4: validation of the scoring system by assessing the reliability of the histological classification prediction

The classification performance of non-experts (n = 3; dentists) is depicted according to the expert ratings (Table 6). Sensitivity and specificity in diagnosing OSCCs as well as the positive predictive and negative predictive value for predicting a histopathological OSCC diagnosis were also calculated for each non-expert. The inexperienced evaluators’ averaged sensitivity was 0.973 and the specificity was 0.881. The weighted Fleiss´ kappa coefficient for multi-rater analysis is also shown in Table 3. Non-experts, using the score sheet, yielded a good agreement value of 0.814 in their diagnostic performance. In order to assess the performance of the score sheet in contrast with the subjective evaluation by the experts, the intra-rater reliability (Table 4) was computed for one inexperienced examiner in a second step. The intra-individual agreement of the non-expert using the score sheet for the analysis of the CLE sequences was very good with a Cronbach’s alpha of 0.884 and an intraclass correlation of 0.792 (95 % confidence interval between 0.703 and 0.856).

**Table 6:** CLE classification performance of the inexperienced examiners (evaluation with newly developed score sheet)

Non-expert	Sensitivity %	Specificity %	PPV %	NPV %
1	100 (50/50)	86.7 (39/45)	89.3 (50/56)	100 (39/39)
2	98.0 (49/50)	80.0 (36/45)	84.5 (49/58)	97.3 (36/37)
3	94.0 (47/50)	97.8 (44/45)	97.9 (47/48)	93.6 (44/47)
Averaged	97.3 (146/150)	88.1 (119/135)	90.1 (146/162)	96.7 (119/123)

*PPV* positive predictive value, *NPV* negative predictive value

## Discussion

In this study we have shown that expert differentiation between physiological and cancerous tissue in the human oral cavity with the help of chair-side CLE examination and subsequent assessment is a valid diagnostic tool with very good results compared to the histopathological evaluation.

Furthermore, we were able to ascertain that non-experts in the field of CLE were able to make a correct diagnosis of OSCC by using the newly proposed classification and scoring system with a classification performance similar to the ratings by experts and the histopathological analysis.

The evaluation of CLE sequences is subjective and a crucial factor in making a correct assessment that requires, up to now, the experience and expertise of specialists. For that reason, and additionally as the mortality of patients critically depends on the tumor stage during the initial diagnosis [29], there is a need for a more standardized CLE evaluation for an early and accurate diagnosis of OSCC that is effective and easily applicable even for non-experts. To reach the aim of a valid and reproducible assessment in daily clinical practice, especially for examiners that are not extensively trained in the field of CLE, it was important to develop a straightforward criteria list and scoring system. To proof the validity of our newly developed scoring system (*DOC*-*Score*), we statistically analyzed non-expert ratings in the differentiation of “physiological” and “cancerous” tissue and compared it to the results achieved by experts and the histopathological assessment.

To the best of our knowledge, there is not yet a standardized procedure for the evaluation of OSCC-CLE sequences. In our study, five criteria were defined, taking into account the findings and expertise of our workgroup in the field of CLE. Furthermore, they were selected by correlating our findings to a number of valuable studies on tissue morphology of squamous cell cancer (SCC) in CLE imaging [3, 13, 23–26, 30].

Squamous cell tissue of the upper aero-digestive tract is known to inherit a specific vessel architecture, consisting of small intrapapillary capillary loops [25, 30]. Deinert et al. [30] described changes in this vessel architecture for early SCCs of the esophagus via CLE imaging. Dilated and elongated intrapapillary capillary loops were reported in malignant squamous cell tissue as a fundamental parameter for tissue characterization in this study. We have found similar results in our study for SCCs of the oral cavity and thus included the vessel morphology as one main criteria in our CLE scoring system (Fig. 4). Prior studies concerning CLE imaging of SCCs focused on specific tissue patterns and cellular components that are characteristic for malignancy [25, 31]. Liu et al. [25] identified dark epithelial cells with irregular arrangement as distinctive for SCCs of the esophagus. These characteristic changes in malignant mucosa are in accordance with OSCCs as we have found in the present study. As the study on tissue architecture as well as cell morphology is a main aspect in CLE-imaging and their alterations are found as being typical in SCCs, two evaluation criteria—namely “tissue architecture” and “cell morphology”—have been included in our scoring system. Haxel et al. investigated one patient with a carcinoma of the floor of the mouth [13] and noticed, besides irregular cell patterns and extended capillaries, a leaking of contrast agent in confocal images. Additionally Nathan et al. [23] described a disorganized epithelium with fluorescein leakage in CLE images in one case of squamous cell carcinoma of the tongue. Our findings, concerning increased fluorescence leakage in SCC, are in accordance to these findings. Therefore we integrated the fluorescein leakage as a further main criterion into our *DOC*-*Score*.

Thus, the following five parameters were defined in our *DOC*-*Score* sheet: 1. *tissue architecture* (1.a homogeneity, 1.b intercellular gaps), 2. *cell morphology*, 3. *fluorescein leakage* and 4. *vessel morphology*. This set of criteria was reviewed and adjusted in multiple discussion rounds and has been strengthened through statistical methods as well as correlation modeling to test the suitability of these items. Exemplary, histological images and their corresponding CLE images are illustrated in Figs. 2, 3 and 4.

Basically, as more dysplastic manifestations get higher scores, a high total sum indicates OSCC when a certain threshold is reached (Table 1, *DOC*-*Score*). This threshold was adjusted and validated according to expert ratings. For this purpose, video sequences not easily or clearly assessable were used by the experts to perform some exemplary calculations in order to determine the proper threshold.

Our final statistical analysis showed that the classification performance of inexperienced professionals was comparable to the subjective diagnoses by experts if the preconditions of (a) an introduction in the field of CLE imaging by standardized slide shows and (b) a rating based on our newly developed score sheet were given. Furthermore, a slightly better inter-rater agreement by laypersons was yielded when applying the score sheet. Thus, good standardization and comparability of the CLE assessment procedure can be ensured for non-experts as well. The intra-rater reliability between the first and the second assessment of the images was better for experts than for non-experts. This suggests that, in our opinion, inexperienced evaluators need reinforcement of the learned lessons at regular intervals in order to maintain the positive learning effect and thus maintain a high level of accuracy in their diagnoses. In spite of the valuable promising results for non-experts, one has to mention that (a) data collection i.e. the handling of the CLE measuring unit and (b) the detection and selection of representative sequences is an integral part of CLE examinations. Both parts were conducted by experts in this study. The performances of the inexperienced examiners in this field of CLE examinations (data collection and sequence selection) were not subject of the present study.

In general, CLE for the imaging of bio-tissue on a microscale has yet to be applied in various medical disciplines including urology, pulmology, dermatology and gastroenterology with good prospects [14, 16, 17, 28]. Recent studies showed promising results regarding a transfer of this imaging method to the area of the head and neck with a sufficient differentiation of pathological lesions to physiological tissue in the upper gastrointestinal tract like pharynx, larynx and the esophagus [3, 24–26]. Furthermore, preliminary results regarding CLE examinations in the oral cavity showed potentials for detecting cancerous lesions even in their early forms. However, these studies were mainly performed either with an experimental set-up, in ex vivo settings, on animal models or with fluorescent agents not suitable for a routine clinical investigation [3, 13, 19–22, 24–26].

A preliminary CLE study has evaluated the identification of non-dysplastic, precancerous and cancerous spots of the oral cavity by experts with high sensitivities of 80–100 %, as well [23]. However, no specific criteria were defined in that study prior to diagnosis regarding the diagnostic process of these entities. Furthermore, Pogorzelski et al. [3] described morphological criteria in CLE imaging of HNSCC regarding the malignant transformation of mucosal tissues. No statistical evaluation of their criteria has yet been performed. Both working groups used experienced investigators as raters.

Our study has some potential limitations: One of them is the number of evaluators (n = 3) who applied our newly developed classification and scoring system in order to validate this score. We generated significant results but there is a need for further validation of the criteria list and scoring system with a greater number of examiners (non-experts) and a larger video database. Furthermore, comparative pictures of apparently physiological tissues were generated in the same patient cohort. Consequently, we cannot completely exclude the possibility, like in similar studies of other groups [23, 32], that these visually identified physiological spots (reference points) could even be degenerated or dysplastic at an earlier stage (no biopsies with histopathological verification). Measuring points of physiological tissue were defined in advance. There is a need to generate pictures of other localizations, like the dorsal tongue (footage is difficult to generate because of the different surface composition [33]) or buccal mucosa. Prospectively, an “optical landscape” of the entire oral cavity should be created, to have the option for comparison in every part of the mouth (for example keratinized versus non-keratinized tissue). Another limitation caused by the technical conditions of pCLE is the limited penetration depth that only enables imaging of superficial mucosa. Submucosal lesion cannot be detected and monitored over time.

In this study we included patients with histopathological confirmed SCCs and differentiated between physiological and malign (OSCC) tissue with the help of the DOC-Score. In our opinion—at the current state of research—this is an essential step that should be built on further. Consequently, there is a need of research work with an enlarged patient size including other entities like precursor lesions of cancer (leukoplakia etc.) or mucosal inflammatory reactions to enable a separation of these entities towards physiological or cancerous mucosa in future.

## Conclusions

In summary, CLE enables a relatively unlimited reproducibly and non-invasive “optical biopsy” for the real-time diagnosis of pathologies. In the area of oral and maxillofacial surgery, it has the potential to reduce the diagnostic time, the morbidity in the sense of an early detection as a screening method and furthermore to identify the exact location and superficial spreading of tumorous mucosa. In order to accomplish these goals, a transfer of the experimental knowledge of CLE imaging in oral and maxillofacial surgery to daily clinical practice through standardized diagnostic procedures is needed. In this study—for the first time in this specialty—with the help of pCLE, an effective and valuable scoring system was developed for the diagnosis of OSCCs. This enables a reliable classification of physiological mucosa and cancerous lesions even for non-CLE-experts in their daily clinical practice. Multicenter studies are now highly warranted to prove our initial findings.
